# Intact Goal-Driven Attentional Capture in Autistic Adults

**DOI:** 10.5334/joc.156

**Published:** 2021-03-26

**Authors:** Layal Husain, Nick Berggren, Anna Remington, Sophie Forster

**Affiliations:** 1 Centre for Research in Autism and Education, Department of Psychology and Human Development, UCL Institute of Education, University College London, UK; 2 Department of Psychological Sciences, Birkbeck College, University of London, UK; 3 School of Psychology, University of Sussex, UK

**Keywords:** autism, selective attention, top-down control, contingent capture

## Abstract

**Background:**

Autistic individuals have been found to show increased distractibility by salient irrelevant information, yet reduced distractibility by information of personal motivational salience. Here we tested whether these prior discrepancies reflect differences in the automatic guidance of attention by top-down goals.

**Methods:**

Autistic (self-reported diagnoses, confirmed with scores on the Social Responsiveness Scale) and non-autistic adults, without intellectual disability (IQ > 80 on Wechsler Abbreviated Scale of Intelligence), searched for a color-defined target object (e.g., red) among irrelevant color objects. Spatially uninformative cues, matching either the target color or a nontarget/irrelevant color, were presented prior to each display.

**Results:**

Replicating previous work, only target color cues reliably captured attention, delaying responses when invalidly versus validly predicting target location. Crucially, this capture was robust for both autistic and neurotypical participants, as confirmed by Bayesian analysis. Limitations: While well powered for our research questions, our sample size precluded investigation of the automatic guidance of attention in a diverse group of autistic people (e.g. those with a range of cognitive abilities).

**Conclusions:**

Our findings imply that key mechanisms underlying the automatic implementation of top-down attentional goals are intact in autism, challenging theories of reduced top-down control.

## Background

Unusual attentional behaviours were referenced in the original definition of autism (Kanner, 1943), and while atypical attention is not a diagnostic feature of autism, it has become increasingly studied as one of the earliest signs of the condition present in infancy (Ames & Fletcher-Watson, 2010). It is important to note, however, that attention in autism is atypical rather than necessarily deficient – having been associated with processing advantages in certain contexts, and proposed as a possible explanation for savant abilities (Ames & Fletcher-Watson, 2010; Remington, Swettenham & Lavie, 2012).

One area in which autistic individuals show both advantages and disadvantages is distractibility – or rather, the automatic and involuntary capture or consumption of attention by stimuli irrelevant to the current task. On one hand, focusing on seemingly irrelevant information in the external environment is, a characteristic behavior in autism (Bryson, Wainwright-Sharp, & Smith, 1990). Objective laboratory measures of distractor processing have also provided evidence of heightened task-irrelevant processing in autism (Christ, Holt, White, & Green, 2007; Remington, Swettenham, Campbell & Coleman, 2009; Remington & Fairnie, 2017). Conversely, however, intriguing recent evidence suggests that certain categories of stimuli which are highly distracting for neurotypical individuals may be more easily ignored by autistic individuals.

One such category is faces, which elicit heightened attentional processing in neurotypical, but not autistic, individuals (Remington, Campbell & Swettenham, 2012). This might be assumed to simply be a consequence of the lack of social prioritization in autism. However, this assumption is challenged by a recent study by Parsons, Bayliss, and Remington (2017), which demonstrated that autistic individuals do not show heightened capture even for categories of stimuli that they are intensely interested in. In this study, both neurotypical and autistic participants with an intense interest in a particular topic (e.g., Harry Potter) engaged in a visual search task for words relating to their topic of interest while ignoring task-irrelevant images related to this topic (e.g., Hogwarts School house logos). While neurotypical adults showed heightened attentional capture by these images, this was not the case for autistic individuals. This finding is particularly striking given that autism is often characterized by intense interests in particular topics (e.g., Turner-Brown, Lam, Holtzclaw, Dichter, & Bodfish, 2011). Paradoxically, despite appearing to focus a lot of attention on these interests in their daily lives, autistic individuals do not appear to automatically prioritize external cues relating to these interests.

The above findings raise the possibility that the mechanism by which attentional priority is allocated to stimuli that we deem personally important may operate differently – perhaps in a less automatic manner – in autistic versus neurotypical individuals. The automatic allocation of attention to stimuli of personal significance has been argued to occur via two routes, the first involving a bottom up ‘self-network’ and the second involving interaction with the executive control system and top-down goals (Humphreys & Sui, 2016; Sui & Rotshtein, 2019). The latter mechanism appears of particular relevance to autism in the light of initial evidence suggesting reduced top-down influences on perception (see Mottron and colleagues’ Enhanced Perceptual Functioning model, Mottron et al., 2006). Likewise, autistic individuals have been shown to be more resistant to visual illusions (Mitchell, Mottron, Soulieres, & Ropar, 2010) and show a general bias towards incoming sensory stimuli rather than relying on prior perceptual experience (Pellicano & Burr, 2012).

While it might appear counterintuitive to suggest that a disrupted top-down attentional mechanism could explain reduced distraction, within the field of selective attention research it is well established that voluntarily focusing attention on a particular type of stimuli can often lead to automatic attentional priority for any stimuli falling within this category. A side effect of this voluntary process is therefore that task-irrelevant stimuli can involuntarily capture attention, even to the detriment of the current task (i.e. cause distraction), if they share defining features of the attended category. To give a real-world example, a bird-watcher looking out for a particular yellow bird might find their attention automatically attracted to a yellow tennis ball. This phenomenon, known as goal-driven, or contingent, attentional capture, has been extensively documented over the past few decades (e.g., Folk & Remington, 1998; Lamy, Leber, & Egeth, 2004; see Eimer, 2014; Olivers, Peters, Houtkamp, & Roelfsema, 2011, for reviews). Although initial demonstrations of goal-driven attentional capture involved top-down goals for simple features such as color, more recent studies demonstrate that goal-driven attentional capture can be induced by adopting a top-down goal for visually diverse semantic categories such as fairground rides or alcoholic beverages (e.g., Brown, Duka & Forster, 2018; Nako, Wu, Smith, & Eimer, 2014; Wyble, Folk & Potter, 2013). As such, goal-driven attentional capture provides a plausible mechanism to explain the automatic prioritization of personally-relevant stimuli – resulting in attentional capture by these stimuli and amplification of associated distractor interference (e.g. response competition interference, see Anderson & Folk, 2014; Berggren, Jenkins, McCants, & Eimer, 2017) – in neurotypical individuals. The goal of the present study was therefore to test the possibility that, for autistic individuals, automatic goal-driven attentional capture may be reduced or even absent.

To date, goal-driven attentional capture has not been studied in autistic adults. However, three studies have examined this phenomenon in autistic children and adolescents, with mixed findings. Greenaway & Plaisted (2005) were the first to investigate this phenomenon, using a classic spatial-cuing reaction time contingent capture paradigm to investigate goal-driven attentional capture in neurotypical and autistic children aged 8–13 (*M* = 11). Their initial experiment showed that adopting a top-down goal for color elicited goal-driven attentional capture among neurotypical and autistic children alike, yet adopting a top-down goal for onset (i.e. a stimulus appearing in a previously unoccupied location) elicited goal-driven attentional capture in the neurotypical group alone. However, their second experiment replicated both the presence of color goal drive capture and absence of onset goal-driven attentional capture in the autistic group, but unexpectedly (and in contrast to the first experiment) also found an absence of color goal-driven attentional capture in the neurotypical group.

More recently, Keehn and colleagues (Keehn et al., 2016; 2017) examined behavioral and neural correlates of goal-driven attentional capture in autistic and non-autistic adolescents, aged 12–17 (*M* = 14). These studies used a rapid serial visual presentation paradigm with a top down goal defined by color, in which contingent capture is typically reflected by increased error rate in association with goal-matching distractors (Serences et al., 2005; Folk et al, 2002). However, neither of these two studies replicated the distracting contingent capture effect on error rate among either the neurotypical or autistic adolescents – a fact the authors attribute to changes made to the task in order to adapt it to the age group. Instead, a facilitatory effect of goal-matching distractors on reaction time was observed, which reached statistical significance only for the neurotypical group. As such, the overall picture regarding the status of goal-driven attentional capture in autism remains unclear, with both prior studies partially replicating established goal-driven capture effects in the neurotypical group, and conflicting conclusions regarding the presence of goal-driven color capture in the autistic group.

The mixed findings from the prior studies could be influenced to some extent by the different paradigms used, but could also potentially be explained by factors relating to the developmental nature of this research: for example, the varying age groups between studies, developmental variation among children of the same age, and developmentally necessary changes made to tasks established in adults. To avoid such factors, the present study compared the degree of goal driven attentional capture in autistic and neurotypical adults, adapting a spatial cueing paradigm that has been well established within adult populations in the study of goal-driven attentional capture (e.g., Eimer, Kiss, Press, & Sauter, 2009; Folk & Remington, 1998). As in the prior work in autistic adolescents and children, we specifically examined contingent capture relating to a color goal. Autistic and neurotypical adults searched displays of colored rectangular bars for a specific target color item (e.g., the blue bar), responding to that object’s orientation. As search displays contained a number of different colored objects, this procedure strongly encourages participants to adopt a specific goal to focus attention on the target color (Bacon & Egeth, 1994). Prior to each search display, a task-irrelevant color cue was presented in one of the potential target locations. This could be either the target color (e.g., blue), a color matching one of the nontarget color bars in the search display, or a wholly irrelevant color. Within this task, goal-driven attentional capture is reflected in a spatial bias to the location of cues that match the attentional goal (i.e. target color). This bias is indexed by slower reaction times when the cue is presented in invalid location (i.e., a location where the target does not then appear) versus when the cue validly predicts the location of the target. The wholly irrelevant color condition allowed cue costs (nontarget-irrelevant) and cue benefits (target-irrelevant) to be established for each participant.

In line with the established patterns using this type of task, we predicted that color cues matching the target color should elicit evidence of attentional capture, as evidenced by reaction time differences following invalid versus valid cues, while no such capture should occur for color cues that do not match the target. Crucially, if autism is associated with reduced evidence of goal-driven attentional capture in comparison to a neurotypical adult sample, target color cues should bias attention less readily for this group.

## Methods

### Participants

Power analysis using G*Power software (Faul, Erdfelder, Lang, & Buchner, 2007) indicated that a total sample size of 32 (16 per group) would have power of β = .97 (α = .05) detect the effect size of the key interaction revealed in Parsons et al’s study (η_p_^2^ = 0.36). We were also well powered (power of β = .80) to detect within-subject effects above *dz* = .75 in each group, which compares favorably with the range of those observed by published demonstrations of goal-driven capture (e.g., Folk et al., 2002: mean *dz* = 1.50, *SD* = .31). To allow for potential exclusions and matching, initial recruitment for the study consisted of nineteen autistic adults and thirty non-autistic neurotypical adults. Neurotypical participants were largely recruited using the University College London’s psychology subject pool, and took part in exchange for course credits. Autistic participants were recruited by emailing adults who were previously involved in studies at the Centre for Research in Autism and Education with information on the project, as well as through various autism charities and social media groups. An incentive of £10 or shop vouchers was given in exchange for participation. All autistic participants reported having a clinical diagnosis of Autism Spectrum Disorder. No participants, in either the neurotypical or autistic group, reported any past or current diagnosis of additional neurological or developmental conditions (e.g., ADHD, dyslexia, epilepsy). The Social Responsiveness Scale (SRS-2: Constantino and Gruber, 2012) was used to confirm diagnoses of the autistic participants and check for undiagnosed cases in the neurotypical group. The measure consists of 65 statements, across six sub-scales, which are self-ranked concerning one’s social behavior over the past six months. The scale has excellent test-retest reliability (.88–.95) and an interrater reliability of (.61–.92) and good internal consistency (α = .95; Bruni, 2014). An SRS t-score over 59 suggests a potential undiagnosed case of autism, and two neurotypical participants were excluded from the study on this basis, as was one autistic participant for scoring in the typical range (*t* < 59). Two further autistic participants were also excluded: one due to below chance-level accuracy in the main task, and one due to being over three standard deviations older in age than the rest of the sample. The final autistic sample therefore consisted of 10 males and 6 females (mean age: 30 years, *SD* = 8). To provide an appropriate comparison, 16 neurotypical individuals were selected on the basis of matching age, gender, and non-verbal IQ (see below) as closely as possible. Associated data from the experimental task was kept blind as part of this process. The final neurotypical sample consisted of 10 males and 6 females (mean age: 26 years; *SD* = 6). See ***Table 1*** for group scores.

**Table 1:** Corresponding mean, standard deviation, and range values for participant age and scores on intelligence tests and the social responsiveness questionnaire (standard deviations in parentheses). Data is split by Group.


		AGE (YEARS)	WASI			SRS-2

			VOCABULARY	MATRIX REASONING	FULL IQ	

Neurotypical	Mean	26(6)	54(10)	61(10)	114(15)	50(5)
Group	Range	19–38	39–72	46–78	90–138	41–58
Autism	Mean	30(8)	62(10)	60(10)	119(14)	75(9)
Group	Range	20–52	43–80	44–74	97–146	61–98


Comparing groups, there was no significant difference in age (*t*(30) = 1.60, *p* = .12). As expected, there was a significant difference in SRS score, with higher scores in the autism group (*M* = 75 vs. 50; *t*(30) = 9.48, *p* < .001).

IQ scores were collected using the Wechsler Abbreviated Scale of Intelligence vocabulary and matrix reasoning subtests (Wechsler, 2011), to obtain a verbal and non-verbal measure of cognitive intelligence for each participant (see ***Table 1***). All participants achieved an IQ score over 80, based on the two WASI subtests of matrix reasoning and vocabulary. Given impairments in verbal ability often seen for autistic individuals, and the non-verbal nature of our task, matching was done on non-verbal IQ only but we report both for information. While there was no significant difference between autistic and neurotypical groups on overall IQ score (*M* = 119 vs. 114 respectively; *t* < 1), and no difference for matrix reasoning (*M* = 60 vs. 61; *t* < 1), a difference was observed for vocabulary, where the autistic group in fact unexpectedly showed a significantly higher score (*M* = 62 vs. 54; *t*(30) = 2.06, *p* = .048). Anecdotal evidence based on experimenter observations suggest that this may have been driven by a somewhat higher proportion of non-autistic individuals who were non-native English speakers. Given that the experimental task in this study did not involve verbal components, these participants were not excluded.

### Materials and Stimuli

**Figure 1:**
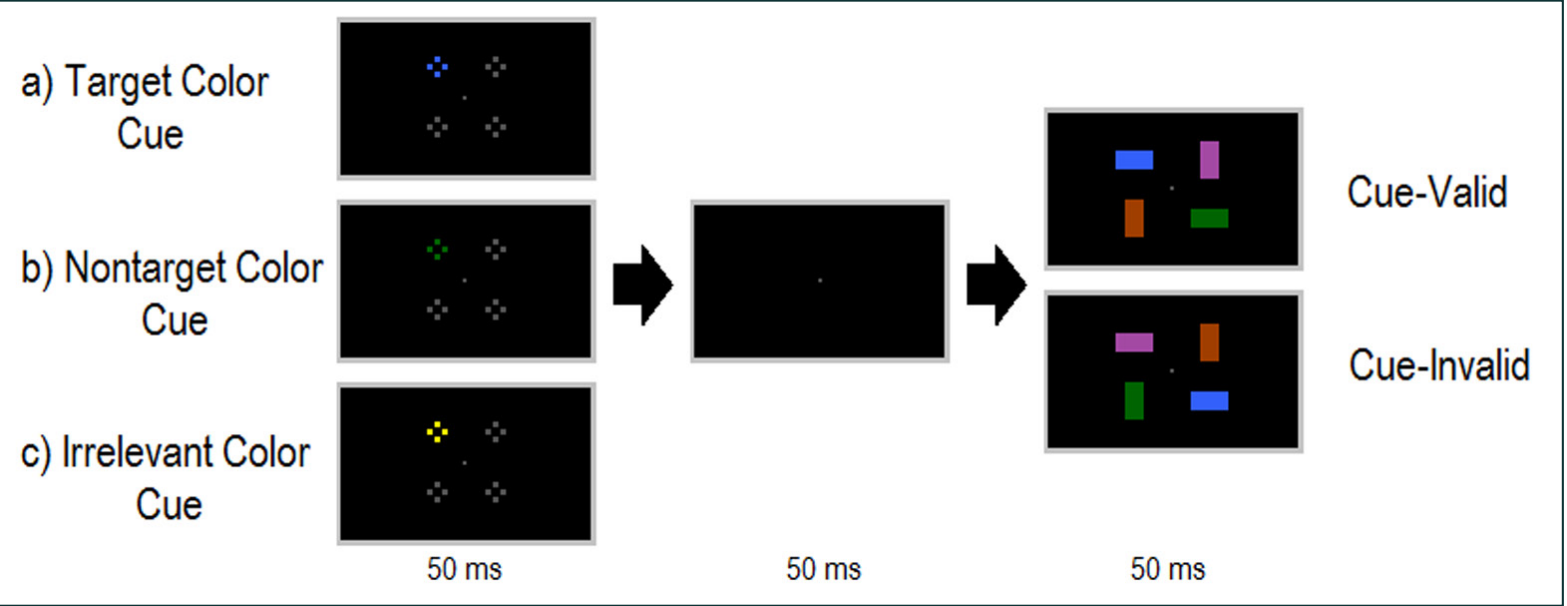
Example experimental trial sequence (not to scale). Participants’ task was to locate a specific color rectangular bar (in this example, blue) and indicate whether this bar was horizontally or vertically oriented. Before each search display, participants viewed one of three possible kinds of cue display: **a)** cue matched the color of the subsequent rectangular bar being searched for (Target Color Cue), **b)** cue color appeared in subsequent search display as a nontarget bar (Nontarget Color Cue), **c)** cue consisted of a color that never appeared in search displays at all (Irrelevant Color Cue). Color cues could either validly or invalidly predict the location of the upcoming target item.

The experimental task was created and run using E-Prime 2.0 software (Psychology Software Tools, Inc.). Stimuli were presented on a 13.3-inch Toshiba laptop (60 Hz; 1366 x 768 screen resolution) at a participant viewing distance of approximately 30 cm. Task responses were recorded via button presses on the laptop keyboard. All stimuli were presented on a black background, with a small grey fixation dot appearing constantly during trials (see ***Figure 1***). Cue displays contained four ‘clusters’, each comprised of four small colored squares, with each small cue measuring 0.38 x 0.38 degrees of visual angle, within each overall ‘cluster’ (1.53 x 1.53°). These clusters appeared in the four quadrants of the screen, equidistant from fixation, at an eccentricity of 2.86° measured from the center of each cluster to fixation. On each trial, three of the clusters contained grey squares, and one contained colored squares. The colors used in the experiment (RGB values in parentheses), were orange (160,62,0), yellow (122,112,0), blue (51,95,250), green (0,100,0) magenta (175,56,255), and grey (110,110,110).

Search display stimuli consisted of four colored rectangular bars (1.15 x 2.67°), presented either in a horizontal or vertical orientation, and at the same locations as cue clusters. Two bars always appeared horizontally and two vertically within each trial, randomly assigned to the four item locations. For each participant, a specific color was randomly chosen as the target, nontarget, and irrelevant color of the cue (see ***Figure 1***). These colors did not vary from trial to trial for an individual participant.

### Procedure

Participants were instructed to search for a specific color of rectangular bar and respond to its orientation, using the ‘A’ key for horizontal and ‘S’ key for vertical. Speed and accuracy were emphasized. ***Figure 1*** presents an example experimental trial. Each trial began with an initial 500 ms fixation period, followed by the cue display (50 ms). One of the four cue clusters appeared in color and could either a) match the color of the target (Target Color Cue), b) match a nontarget color that appeared in one of the search displays as a nontarget rectangular bar (Nontarget Color Cue), or c) be an entirely irrelevant color that never appeared within search displays (Irrelevant Color Cue). The color cue was spatially uninformative regarding the location of the upcoming target rectangular bar, and so was thus ‘valid’ on 25% of trials (target appeared in same location as cue) and ‘invalid’ on 75% of trials (target appeared in one of the other three locations). Participants were instructed to ignore the cue displays and maintain fixation on the central grey dot. Following a 50 ms inter-stimulus interval, search displays appeared for 50 ms followed by a blank response screen (1450 ms or until response was made). This short inter-stimulus interval between cue and search displays was chosen to minimize effects of post-capture processes such as disengagement from cues, allowing us to test the magnitude of initial capture by cues based on task goals (see Berggren & Eimer, 2016, Experiment 2). If no response was detected, the trial was counted as incorrect at the end of the response window.

Participants performed a practice block of 12 trials, repeating as required to feel confident with the task and achieve >70% accuracy (no participant repeated more than once). Participants completed six experimental blocks of 96 trials each. The task took around thirty minutes to complete. Each block counterbalanced cue type (3), cue location (4), and target location (4), with all other variables randomized. During experimental blocks, the experimenter could pause the block if requested by participants, however this was never necessary. Opportunities for breaks were given at the end of each block. After completing the experimental task, participants completed and SRS-2 form and IQ measures as described above.

## Results

*Reaction Times:* Median response-accurate reaction time (RT) data were entered into a 3x2x2 mixed Analysis of Variance (ANOVA) with the factors Cue Type (Target Color Cue, Nontarget Color Cue, Irrelevant Color Cue), Cue Validity (Cue-Invalid, Cue-Valid), and Group (Neurotypical, Autistic). See ***Table 2*** for group averages. Analysis showed a significant main effect of Cue Type (*F*(2,60) = 4.21, *p* = .02, η_p_^2^ = .12). Pairwise comparisons of each Cue Type level suggested that this effect was due to generally slower response times on trials that contained an irrelevant color cue (*M* = 536 ms), compared to target (*M* = 528 ms; *t*(31) = 3.02, *p* = .005) and nontarget color cues (*M* = 529 ms; *t*(31) = 2.12, *p* = .04), which did not differ (*t*(31) < 1.0, *p* = .61). There was also a significant main effect of Cue Validity (*F*(1,30) = 18.05, *p* < .001, η_p_^2^ = .38), with reaction times slower on cue-invalid than cue-valid trials (*M* = 537 vs. 525 ms).

**Table 2:** Median reaction time (RT) in milliseconds and mean error rate (percentage incorrect trials) across Cue Type and Cue Validity conditions (standard deviations in parentheses). Data is presented overall and split by Group.


		TARGET COLOR CUE		NONTARGET COLOR CUE		IRRELEVANT COLOR Cue	

		CUE- INVALID	CUE- VALID	CUE- INVALID	CUE- VALID	CUE- INVALID	CUE- VALID

All participants	RT Error rate	554 (59) 5 (5)	501 (53) 3 (4)	526 (55) 3 (3)	532 (61) 3 (4)	531 (56) 4 (4)	541 (62) 4 (5)
Neurotypical Group	RT Error rate	534 (54) 4 (5)	483 (50) 2 (3)	508 (51) 3 (2)	509 (56) 3 (4)	512 (54) 3 (2)	519 (60) 2 (2)
AutismGroup	RT Error rate	575 (59) 6 (6)	519 (51) 3 (5)	545 (53) 4 (4)	555 (59) 4 (4)	551 (53) 4 (5)	563 (58) 5 (6)


**Figure 2:**
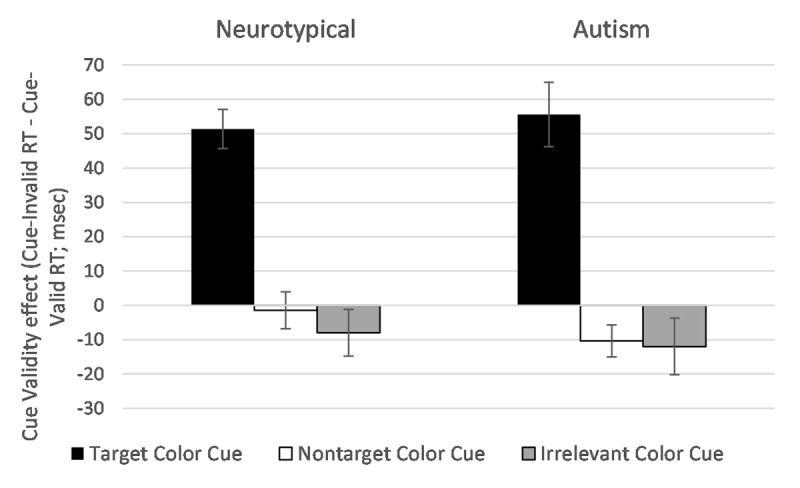
Cue validity effects on reaction time (calculated as Cue-Invalid minus Cue-Valid RT in milliseconds) across each Cue Type condition, separately for neurotypical and autism groups.

Consistent with established contingent capture effects, there was a significant Cue Type x Cue Validity interaction (*F*(2,60) = 56.05, *p* < .001, η_p_^2^ = .65). As can be seen in ***Figure 2***, this reflected that significant attentional capture (indexed by the cue validity effect: RT difference in the presence of valid versus invalid cues) was found only when the cue color matched the current top-down search goal. Target color cues (i.e. those matching the current top-down goal) elicited a robust cue validity effect suggesting attentional capture (*M* diff = 53 ms; *t*(31) = 9.88, *p* < .001). In contrast, nontarget color cues did not appear to capture attention (*M* diff = –6 ms; *t*(31) = 1.65, *p* = .11). For irrelevant color cues, there was if anything evidence of a reversed cue validity effect (*M* diff = –10 ms; *t*(31) = 1.90, *p* = .067). In relation to Group, there was a significant main effect (*F*(1,30) = 4.84, *p* = .04, η_p_^2^ = .14), with generally slower RTs among the autistic group (*M* = 551 vs. 511 ms). It is important to note that generally slower RTs in the autism group have been found in a number of motor response studies (e.g., Baisch, Cai, Li, & Victor Pinheiro, 2017), and does not necessarily reflect a difference in visual attention (in contrast to derived RTs in relation to the validity of spatial cues). There was no evidence to suggest that Group interacted with Cue Type (*F*(2,60) < 1.0, *p* = .88), Cue Validity (*F*(1,30) < 1.0, *p* = .63), or as part of a three-way interaction (*F*(2,60) < 1.0, *p* = .61).

***Figure 2*** shows average cue validity effects within each level of Cue Type separately for neurotypical and autism groups for illustrative purposes. Crucially, target color cues elicited robust attentional capture of similar magnitude in both groups (mean cue validity effects were 51 ms and 56 ms for the neurotypical and autistic groups respectively; *t*(30) < 1.0, *p* = .70).

A Bayesian analysis was conducted to further assess the sensitivity of this null result. A Bayes Factor (B) > 3 demonstrates substantial evidence for H1, while B< 0.33 indicates substantial evidence for H0, with Bayes factors between these values suggests insensitivity (Dienes, 2014). Given our directional hypothesis, a Bayes factor was calculated using a half-normal distribution with a mean of zero. The prior was the 76ms difference observed by Parson and colleagues (2017) between the attentional capture effects among neurotypical and autistic adults. Contrasting the respective goal-driven attentional capture effects (i.e. the validity effect for target color cues) for autistic versus neurotypical adults revealed sensitive evidence for the null hypothesis of no group differences in goal-driven attentional capture, B_H(0, 76)_ = 0.15.

*Error rates:* While RT is the primary measure within this paradigm, an equivalent ANOVA was conducted on error rate data (see ***Table 2*** for overall sample and group accuracy rates). This showed no significant main effect of Cue Type (*F*(2,60) < 1.0, *p* = .66) or Cue Validity (*F*(1,30) = 2.74, *p* = .11). There was, however, a significant Cue Type x Cue Validity interaction (*F*(2,60) = 4.06, *p* = .02, η_p_^2^ = .12). Consistent with the RT data, cue validity effects on error rates only occurred following Target color cues (*M* diff = 1.81 %; *t*(31) = 3.34, *p* = .002), with no difference in Nontarget (*M* diff = .16 %; t(31) < 1.0, *p* = .72) or Irrelevant color cue conditions (*M* diff = –.19 %; *t*(31) < 1.0, *p* = .79). Finally, there was no overall main effect of Group (*F*(1,30) = 1.36, *p* = .25), and Group did not reliably interact with Cue Type (*F*(2,60) = 1.54, *p* = .22), Cue Validity (*F*(1,30) < 1.0, *p* = .63), or as part of a three-way interaction (*F*(2,60) = 1.55, *p* = .22).

## Discussion

To address conflicting findings that autistic individuals show increased processing of salient irrelevant information (e.g. additional auditory scene components, Remington & Fairnie 2017), but paradoxically reduced distraction by personally-relevant salient information (e.g. pictures related to intense interests, Parsons et al., 2017), the present study investigated the possibility that goal-driven attentional capture typically seen in neurotypical individuals may be reduced or absent in autism. Contrary to our hypothesis, robust goal-driven attentional capture, as reflected by biased attention towards target color cues, was observed in autistic and neurotypical participants alike. This implies that, at least in this instance, the automatic guidance of attention in accordance with voluntary attentional goals operates similarly in autistic versus neurotypical adults.

Our findings also demonstrate no difference in the magnitude of goal-driven attentional capture by target color cues for the autistic and neurotypical participants. Rather, both groups showed clear evidence of such capture, with Bayesian analysis providing sensitive evidence for a null difference. Moreover, the within-subjects cueing effect observed for both groups in the present study (approximately 60 ms slower for cue-invalid versus cue-valid trials) is consistent in magnitude with previous work on attentional capture in the general population (e.g., Berggren & Eimer, 2016). This rules out the possibility that the neurotypical participants in our study were displaying unusually low attentional capture. As such, we offer evidence that, at least in certain situations, autistic individuals show automatic attentional capture based on the voluntary goals of the task in question.

Our findings extend prior work which has led to mixed conclusions regarding whether goal-driven color capture remains intact in autistic children and adolescents (Greenaway & Plaisted, 2005, Keehn et al., 2017, 2016). Our pattern of results is consistent with the findings of Greenaway and Plaisted (2005), which like the present study used a spatial cuing measure of goal-driven attentional capture. Taken together with Greenaway and Plaisted’s findings in children, our results suggest that goal-driven attentional capture, at least for static features such as color, can remain intact in autism from mid childhood to adulthood. In considering why Keehn and colleagues revealed a different pattern of behavioral performance among autistic adolescents, one relevant factor may be the different paradigms used between studies. Both Greenaway and Plaisted and the present study used spatial cuing measures of contingent capture, while Keehn and colleagues adapted a different contingent capture measure that typically involves a more temporal form of disruption manifesting on error rates. Both the present study and the study by Greenaway and Plaisted replicated the goal-driven capture effects typically observed in our spatial cuing paradigms, and based conclusions on these effects. Keehn and colleagues, however, did not replicate the expected pattern of capture effects from their RSVP paradigm and instead based their conclusions on an intriguing faciliatory effect. As such, one possibility is that the facilitatory effect observed by Keehn and colleagues might reflect a distinct mechanism from the spatial cuing capture observed in the present study (e.g. priming associated with non-spatial processing of the distractor identity).

More generally, our finding of intact goal-driven attentional capture in autistic adults challenges the hypothesis that autistic individuals’ attentional pathways are minimally altered by top-down influences (Mottron et al., 2006; Maekawa et al., 2011). If this were the case, one would expect a reduced ability to adopt and maintain a color-specific search goal in order to guide attention. This observation also more generally contrasts with a number of studies that have demonstrated what appears to be a lesser influence of past experience on perceptual processing (see for example the work on reduced adaptation effects by Pellicano et al., 2007; Turi et al., 2016). However, the situation is by no means clear-cut. In other situations, typical adaptation effects are seen in autistic individuals (Intaité et al., 2019). Taken together, Palmer et al. (2017) suggest, in their recent and thorough review of the topic, that perceptual atypicalities may vary as a function of 1) type of stimulus (social vs. nonsocial), 2) sensory modality (auditory vs. visual), 3) age of participant and 4) complexity of stimulus (high vs. low level of processing).

When considering the discrepancy between intact goal-directed capture (current study) and reduced impact of interest and expertise on attentional capture (Parsons et al., 2017), it should be noted that the use of non-social stimuli, modality and age of participant remained constant between the two studies. These factors can therefore not explain the disparity in findings. However, participants were somewhat older in the present study (*M* = 30), compared to the Parsons study (*M* = 19), with the latter study including both adolescents and adults. Another potentially important difference between the Parsons study and the present work may be that the lower level of processing required in the study presented here allows for evident top-down influences. To explore this possibility further, future research should aim to use more complex stimuli (photographs similar to those in Parsons et al., 2017) in a contingent-capture paradigm.

Regardless of the above differences, it is clear that our results do not support a simple goal-driven account of the reduced capture by personally-relevant stimuli. As such, future work should examine alternative explanations for the differences observed by Parsons et al. (2017), such as disruptions to the bottom-up self-network (Humphreys & Sui, 2016; Sui & Rotshtein, 2019). Another possibility is that the impact of time on top-down influences is different in autistic and non-autistic individuals. When considering reduced automatic attentional priority for items related to topics of interest in autistic individuals (Parsons et al., 2017), this expertise and interest had been previously established over months and years, rather than a temporary color goal established in minutes. Indeed this distinction between short and long range timescales was recently proposed to be crucial in understanding the nature of perceptual atypicalities in autistic individuals. Palmer and colleagues (2017) remark on the diversity of performance on attentional paradigms that span various stimulus types, spatial and temporal scales. For example, the resistance to visual illusions seen in autistic individuals is highlighted as evidence for a persistent reduction in top-down influences over longer time periods (Mitchell et al., 2010), in contrast to work by Manning and colleagues (Manning, Kilner, Neil, Karaminis, & Pellicano, 2017) that demonstrated equivalent modification of behavior by autistic and non-autistic children in response to changing stimulus reward contingencies across a short experimental task. This may be akin to our dissociation between the lack of influence of long-term interest and expertise on automatic processing of distractor images for autistic individuals (Parsons et al., 2017) and the findings of intact top-down goal-directed behavior by autistic participants in the present study.

As such, we must now establish whether these goal-related alterations in stimulus saliency are maintained over the longer term (an area that is understudied even in neurotypical populations). For example, it is believed that short-term goals established to aid the guidance of attention are held within visual working memory, but that sustained goals migrate to a longer term store (e.g., Berggren & Eimer, 2018; Woodman, Luck, & Schall, 2007). The precise mechanisms behind the guidance of attention by long-term motivations and goals, and their qualitative difference to short-term goals, could offer great insight into atypical top-down attention processes in autism. Our results nonetheless provide a first step towards identifying more specifically where in the process of goal-driven attention differences might occur.

Overall, it is also of note that our findings add an additional data point to the collection of studies that reveal intact or even superior performance of autistic individuals on tasks of attention and perception. While there is now growing acceptance that autism is associated with strengths as well as challenges, historically a more negative view was taken. There are many instances of differences that are interpreted as deficits, even when behavioral results do not support this conclusion (Dinishak, 2016). The current results therefore complement the implication for clinical and educational practices in autism that altered performance observed under some task contexts do not necessarily imply broad deficits in attentional control.

## Limitations

While our sample size was highly powered to detect our within and between subject effects of interest, and in line with sample sizes from similar experimental paradigms with this clinical population (e.g. Christ et al, 2007; Parsons et al., 2017; Keehn et al., 2016), it did not allow us to consider diversity within those on the autistic spectrum. For example, only those without co-occurring intellectual impairment participated in this study. Given that many autistic people have intellectual difficulties (an estimate of up to 50%, Charman et al., 2011) we must be cautious about generalizing the present findings to the whole autistic population. Future research with larger sample sizes could address this, considering in particular a wider range of cognitive abilities, and the role of ADHD (which commonly co-occurs with ASD and involves attentional differences).

## Conclusions

The present study demonstrates that a key attentional mechanism underpinning the automatic implementation of top-down attentional goals functions similarly in neurotypical adults and autistic adults without intellectual impairment. As outlined above, previous evidence has pointed to both increased and reduced distractibility in autistic individuals. Here we delineate one of a number of forms of distraction, automatic goal-driven attentional capture, finding that this process appears entirely preserved in autistic individuals. These results provide an additional benchmark for future research to pinpoint the precise locus of attentional differences seen as a fundamental aspect of autistic symptomatology.

## Data Accessibility Statement

Participants did not give permission for the dataset to be published, and therefore we are not able to provide a public link to the data. However, anonymized versions of the datasets used during the current study are available from the corresponding author on request.
